# Facial Gender-Confirmation Surgery: A Systematic Mapping Review of Surgical Growth and Outcome Gaps

**DOI:** 10.3390/medicina62071303

**Published:** 2026-07-06

**Authors:** Gustavo Sáenz-Ravello, Paula Carrasco García, Shane D. Morrison, Alberto Inzulza Galdames, Thais Calderon, Elie M. Ferneini, Elda L. Fisher

**Affiliations:** 1 School of Health and Wellbeing, Health Economics and Health Technology Assessment, University of Glasgow College of Medical Veterinary and Life Sciences, Glasgow G12 8QQ, UK; 2 Servicio de Salud Tarapacá, Ministerio de Salud, Iquique 1130000, Chile; 3 Division of Plastic Surgery, Department of Surgery, Seattle Children’s Hospital, Seattle, WA 98145, USA; 4 Subsecretaría de Salud Pública, Gabinete, Ministerio de Salud, Santiago 8320064, Chile; 5 Departamento de Tecnologías de Gestión, Facultad Tecnológica, Universidad de Santiago de Chile, Santiago 9170022, Chile; 6 Ferneini Maxillofacial Surgical Institute, Cheshire, CT 06410, USA; 7 Beau Visage Med Spa, Cheshire, CT 06410, USA; 8 Department of Surgery, Frank H. Netter MD School of Medicine, Quinnipiac University, North Haven, CT 06473, USA; 9 Division of Oral and Maxillofacial Surgery, University of Connecticut School of Dental Medicine, Farmington, CT 06030, USA; 10 Division of Plastic, Maxillofacial, and Oral Surgery, Department of Surgery, Duke University Medical Center, Durham, NC 27710, USA

**Keywords:** facial gender-confirmation surgery, facial gender-affirming surgery, facial feminization surgery, facial masculinization surgery, transgender surgery, systematic review

## Abstract

*Background and Objectives*: Facial gender-confirmation surgery (FGCS) has expanded rapidly but remains uneven across procedures, specialties, and outcome domains, limiting clinically useful interpretation for surgeons. The aim is to characterize how FGCS evidence has developed and to identify gaps relevant to surgical reporting and patient-centered outcome evaluation. *Materials and Methods*: Systematic searches were conducted without date restriction in PubMed, Scopus, Web of Science, LiLACS, and EBSCOhost through June 2025. Eligible studies included original clinical research, systematic reviews (SR), technical notes, and economic evaluations. Two reviewers independently screened and extracted data. PROSPERO: CRD420251041192. *Results*: Among 338 included studies, the literature was concentrated on feminization procedures (78%) and hard-tissue interventions (51%). Outcome reporting was dominated by surgical technique (36%) and complications (29%); patient-centered domains were less represented, including satisfaction (25%), preferences (7%), recovery (2%), and ethics (3%). Masculinization and soft-tissue procedures were less frequently studied. Methodological quality of the 36 included SRs was predominantly critically low (72%) or low (28%). *Conclusions*: Procedural reporting in FGCS has developed asymmetrically relative to standardized patient-centered outcome measurement, a pattern consistent with the formative stages observed in other surgical fields. For surgeons, this limits comparability across procedures and weakens the evidence base for counseling, multidisciplinary planning, and postoperative evaluation. Future research should prioritize more consistent and validated patient-reporting frameworks.

## 1. Introduction

Gender incongruence (GI) is characterized by a persistent discrepancy between an individual’s experienced gender identity and the sex assigned at birth [1] and may give rise to clinically significant psychological distress [2], commonly defined as gender dysphoria (GD) [3]. In response, many trans- and gender-diverse (TGD) individuals pursue gender-affirming interventions across social, legal, hormonal, and surgical domains to achieve greater congruence between their physical characteristics and gender identity. Among these interventions, facial gender-affirming surgery occupies a pivotal role, given the centrality of the face in social interactions and gender recognition [4].

**Figure 1:**
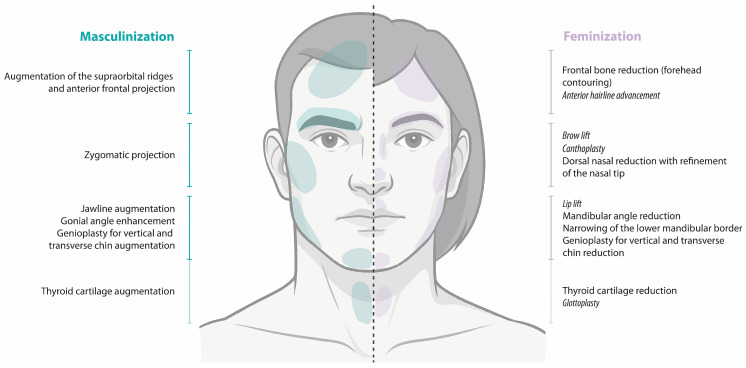
Summary of commonly performed procedures in facial gender-affirming surgery, categorized by upper, middle, and lower facial thirds. Color overlays indicate surgical zones typically involved in masculinization (green) and feminization (lavender) interventions.

Historically discussed as facial feminization surgery, the field has expanded conceptually and procedurally to include a broader spectrum of facial gender-affirming interventions, including masculinization, now more inclusively referred to as facial gender-confirmation surgery (FGCS) [5,6]. FGCS spans multiple facial regions, tissue planes, and specialty interfaces (Figure 1) and includes procedures intended to reduce gender dysphoria. These include osteoplastic maxillofacial, soft-tissue plastic, and adjunctive phonatory procedures, each playing a distinct role in shaping gender perception, facial harmony, and self-identity. Despite this expansion of the literature over the past two decades, inconsistencies in terminology, the absence of standardized surgical protocols, variable referral criteria, and disparities in access to care continue to pose challenges [5]. Moreover, the classification of FGCS as merely “cosmetic” by some health systems and insurance providers persists despite accumulating evidence supporting its role in alleviating GD, improving mental health, and enhancing quality of life [7]. As emphasized [8], these surgeries should be understood within the framework of medically necessary gender-affirming care, consistent with the evolving Standards of Care of the World Professional Association for Transgender Health (WPATH).

In this context, evidence mapping is useful to characterize heterogeneous research fields and identify where evidence is concentrated or remains limited, rather than to simply organize a fragmented literature [9,10]. Accordingly, this mapping review addressed two questions. First, how is the FGCS literature distributed across facial thirds, tissue types, surgical specialties, and outcome domains? Second, what does the methodological maturity of secondary evidence in this field, as assessed by AMSTAR-2, indicate about the current state of synthesis? Together, these questions are intended to characterize the structure and reliability of the published evidence base available to surgeons involved in facial gender-affirming care.

## 2. Materials and Methods

### 2.1. Protocol and Study Design

This study was conducted following the guidance published by expert authors [9,10,11]. The protocol for this mapping review was prospectively registered in PROSPERO (CRD420251041192, date of registration: 27 July 2025) and complies with the PRISMA checklist for Systematic Reviews [12] and PRITEM Reporting Guideline for Mapping Reviews [13]. No amendments to the original protocol were made.

### 2.2. Conceptual Framework and Expert Consultation

Because the FGCS literature is heterogeneous in terminology, the procedural scope and mapping framework were defined before screening. This framework was intended to provide a clinically interpretable structure, outcome reporting, and a priori structured data for understanding how the literature has developed across facial regions, tissue type, surgical specialty, and clinical outcomes in the published literature. A mapping methodology was selected because FGCS research is heterogeneous in terms of design and reported outcomes, making conventional systematic synthesis insufficient to capture field-wide patterns. Studies were eligible if they focused on FGCS and provided extractable information relevant to the predefined classification framework (Table 1). We excluded studies not focused on facial procedures, non-surgical interventions without a facial surgical component, and publications lacking extractable information relevant to the mapping domains.

### 2.3. Information Sources and Selection Process

The literature search was based on PRISMA-S [14] recommendations and was performed independently by two reviewers (GSR, PCG). The databases used were EBSCOhost, LiLACS, PubMed, Scopus, and Web of Science. The sources were consulted for studies published until 6 June 2025, without date restrictions. Search strategies incorporated historical and contemporary terminology, including facial feminization surgery, facial masculinization surgery, facial gender-affirming surgery, and facial gender-confirmation surgery. The algorithms used to conduct the search were developed by an experienced reviewer (GSR), starting from the PubMED thesaurus (MeSH terms) related to FGCS, which were adapted to the other search platforms (See Table S1, Supplementary File). Additionally, we contacted the authors of relevant studies to ask if they were aware of additional studies that may be of interest to our investigation. All retrieved records were imported into EndNote™ (v21.5; Clarivate, Philadelphia, PA, USA) for deduplication. Title and abstract screening were supported using ASReview (v.2.1, ASReview LAB developers, Utrecht University, Utrecht, The Netherlands) [15], with inclusion and exclusion decisions independently confirmed by two reviewers (GSR, PCG). Full-text screening was subsequently performed in Rayyan (Rayyan Systems Inc., Cambridge, MA, USA) [16]. Reference lists of included studies were also screened for additional eligible publications. Excluded studies and reasons for exclusion are presented in Table S2 (Supplementary File). Inter-reviewer agreement was assessed using Cohen’s kappa. Disagreements were resolved by consensus or by consultation with a third reviewer (EF).

### 2.4. Data Collection Process

Initial data extraction was performed by one reviewer (GSR), followed by independent verification of a random 20% sample by a second reviewer (PCG). When conceptual discrepancies arose, data were re-extracted and compared until agreement was achieved. Using a predefined extraction form in Microsoft Excel 2024 (Microsoft Corporation, Redmond, WA, USA), studies were classified according to the structured mapping framework shown in Table 1. Extracted domains included facial third, tissue type, surgical specialty, diagnosis and planning, surgical aspects, post-surgical outcomes, and selected health-system considerations. This approach was intended not only to summarize publication patterns but also to provide a reusable scheme for future evidence synthesis in FGCS. Authors were contacted by email when key information was missing from published reports (two attempts).

### 2.5. Data Synthesis

We synthesized the data following SWiM [17], PRISMA-ScR [18] and SAGER [19] guidance: all study-level variables extracted in Microsoft Excel were imported into Python 3.11 (pandas) (Python Software Foundation, Wilmington, DE, USA) for descriptive analysis. To visualize the structure of the evidence base, we developed interactive evidence map in Dash (Plotly, Plotly Technologies Inc., Montréal, QC, Canada), crossing procedures (rows, grouped by facial third) against evidence domains (columns: gender target, tissue type, surgical specialty, diagnosis and planning, surgical aspects, post-surgical aspects, and health-system considerations). Bubble size represented the number of studies and color indicated study design. Narrative synthesis focused on areas of concentration and comparatively sparse domains across the facial third, gender target, and evidence domain. Duplicate reports from the same cohort were consolidated so that counts reflected unique populations.

Systematic reviews (SR) identified within the corpus were independently appraised by two reviewers (GSR, PCG) using AMSTAR-2 to characterize the methodological maturity and transparency of prior FGCS syntheses [20]. This assessment was conducted for contextual rather than exclusionary purposes. As a mapping review, this study describes published evidence patterns but does not infer adequacy, clinical frequency, or comparative importance of procedures [9].

## 3. Results

**Figure 2:**
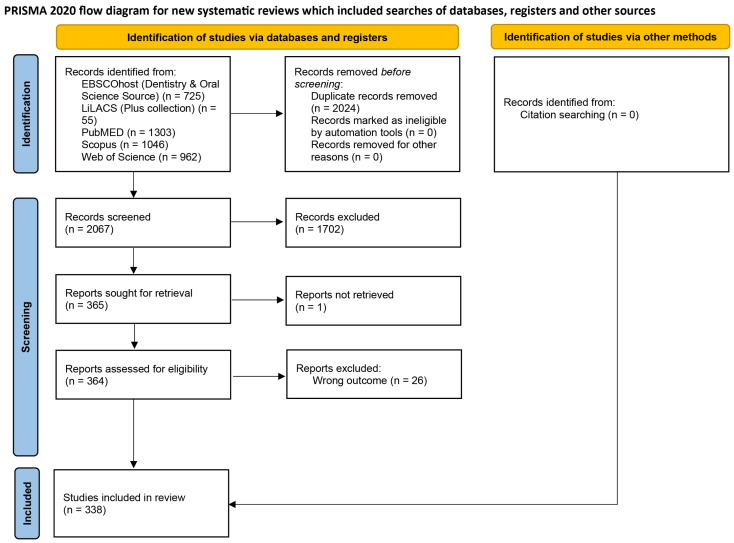
PRISMA 2020 flow diagram showing the systematic review process.

A total of 4901 records were identified through database and registry searching, with no additional studies identified through other sources. After removal of duplicates (n = 2024), 2067 records underwent title and abstract screening, leaving 365 reports for full-text assessment. One report could not be retrieved. Following full-text review, 338 studies were included (Cohen’s kappa = 0.86 during screening; 100% agreement at the full-text stage) (Figure 2). Reasons for exclusion are detailed in Table S2 (Supplementary File).

### 3.1. Characteristics of the Included Studies

Most studies were published between 1996 and 2025 and originated from North America (n= 245, 72.5%), followed by Europe (n = 60, 17.8%), Asia (n = 16, 4.7%), South America (n = 13, 3.8%), and Oceania (n = 4, 1.2%). The literature was strongly weighted toward facial feminization procedures (n = 264, 78.1%), whereas masculinization accounted for a much smaller proportion of studies (n = 67, 19.8%); only seven studies (2.1%) addressed both. Anatomically, nearly half of the included studies involved procedures spanning multiple facial thirds (n = 166, 49.1%), followed by the lower third (n = 74, 21.9%), upper third (n = 55, 16.3%), and middle third (n = 22, 6.5%). A minority referred to full facial gender-affirming surgery without restriction to a specific third (n = 21, 6.2%).

With respect to tissue type, hard-tissue procedures were slightly more common than soft-tissue procedures (n = 172, 50.9% vs. n = 137, 40.5%), while 29 studies (8.6%) included both. In terms of specialty, plastic surgery accounted for most reports (n = 182, 53.9%), followed by otorhinolaryngology and head and neck surgery (ENT-HNS) (n = 76, 22.5%), maxillofacial surgery (OMFS) (n = 36, 10.7%), multidisciplinary teams (n = 17, 5.0%), and other specialties (n = 27, 8.0%).

### 3.2. Study Designs and Outcomes

Cohort studies were most frequent (n = 106, 31.4%), followed by cross-sectional studies (n = 73, 21.6%) and technical notes (n = 70, 20.7%). Smaller proportions corresponded to SR (n = 36, 10.7%), case series (n = 22, 6.5%), case reports (n = 15, 4.4%), pre-clinical studies (n = 6, 1.8%), essays (n = 6, 1.8%), and qualitative studies (n = 4, 1.2%). Outcome reporting was similarly uneven and favored procedure-centered domains. Surgical technique was the most frequently reported outcome domain (n = 120, 35.5%), followed by aesthetic outcome assessment (n = 105, 31.1%), clinical evaluation (n = 101, 29.9%), and complications (n = 97, 28.7%). Imaging-based outcomes were less common, with 3D radiographic analyses reported in 13.6% of studies (n = 46) and 2D analyses in 3.6% (n = 12).

By contrast, patient-centered outcomes were reported less consistently. Satisfaction was evaluated in 24.9% of studies (n = 84), and patient preference in only 6.5% (n = 22). Recovery protocols (n = 7, 2.1%), patient education (n = 8, 2.4%), and revision procedures (n = 19, 5.6%) were also infrequently addressed. Broader structural aspects of care were rarely examined: insurance coverage appeared in 7.4% of studies (n = 25), and ethical considerations in only 3.0% (n = 10).

**Figure 3:**
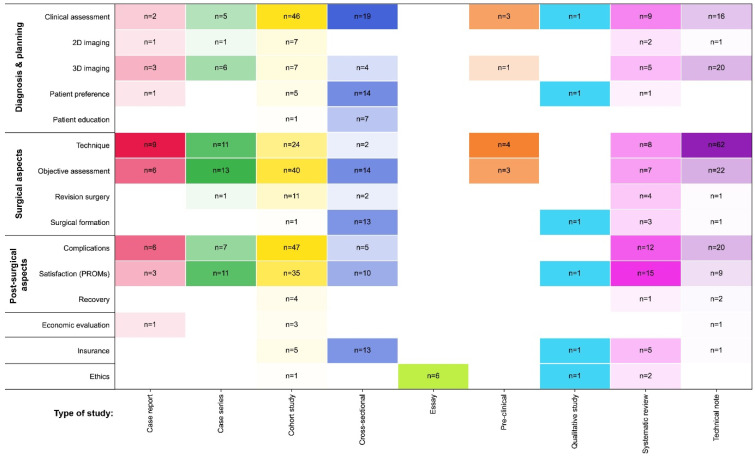
Distribution of studies on facial gender-affirming surgery according to topic and study design. Rows represent thematic categories grouped into diagnostic/planning, surgical, and post-surgical aspects. Columns indicate study design. Cell color denotes study type, and numbers indicate the count of articles in each category.

Figure 3 summarizes the distribution of outcome domains across study designs. Outcomes were grouped into diagnosis and planning, surgical aspects, and post-surgical aspects. Cohort studies contributed the largest number of outcomes across most domains, particularly clinical assessment (n = 46), objective assessment (n = 40), complications (n = 47), and satisfaction (n = 35). Cross-sectional studies also contributed substantially to clinical assessment (n = 19), objective assessment (n = 14), surgical training (n = 13), insurance (n = 13), and satisfaction (n = 10). Technical notes and systematic reviews were concentrated mainly in surgical technique (n = 62 and n = 8, respectively), complications (n = 22 and n = 12), and satisfaction (n = 20 and n = 15). Case series and case reports contributed fewer outcomes overall and were centered largely on technique, complications, and objective assessment. Only a small number of studies, mainly essays and cross-sectional designs, addressed insurance, ethics, or economic evaluation. Across designs, outcomes related to technique, complications, and objective assessment predominated, whereas recovery, patient preference, economic evaluation, and ethics remained sparse.

Temporal visualization of the mapped literature suggested three broad developmental phases in FGCS research: an early phase (1996–2010) dominated by technique-focused reports and case-based publications; a consolidation phase (2011–2021) characterized by expansion in specialty participation and increasing use of imaging-based planning; and a contemporary phase (2022 onward) marked by the emergence of validated patient-reported outcome measures and early multidisciplinary collaboration.

### 3.3. Evidence Map and Research Gaps

**Figure 4:**
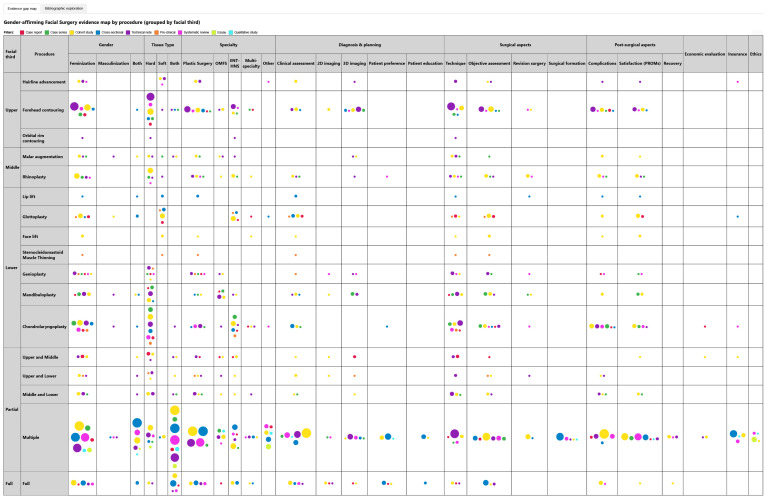
Interactive evidence gap map of facial gender-affirming surgery, organized by procedure and mapped across study characteristics, surgical aspects, and outcomes. Each bubble represents one or more studies, with size proportional to the number of articles and color corresponding to the study type.

The evidence map (Figure 4) showed clear concentration in upper-third procedures, particularly forehead contouring and hairline advancement, which were most often reported in feminization-focused, hard-tissue studies, predominantly within plastic surgery. These studies commonly addressed planning, imaging, clinical assessment, and technique. Middle- and lower-third procedures, including malar augmentation, mandibuloplasty, and glottoplasty, were represented by fewer and more heterogeneous studies across specialties and outcomes. Masculinization was less frequently studied overall and was mainly concentrated in forehead contouring, mandibuloplasty, and multi-region procedures. Across the map, hard-tissue reporting predominated, and the most represented domains were technique, assessment, complications, and satisfaction. In contrast, patient-centered and structural domains, including preferences, education, insurance, economics, and ethics, were sparsely represented. Studies addressing multiple procedures showed the broadest range of reported outcomes (Table 2). An expanded interactive version of the evidence map, including integrated search functions and additional exploratory views of the dataset, is available online, https://pa6t2w-gustavo-s0enz.shinyapps.io/evidencemapfgas/ (accessed on 24 June 2026), The resulting map allows clinicians to identify evidence-supported and underreported procedures and outcomes, researchers to locate gaps and avoid duplication, and guideline developers to distinguish evidence-dense areas from domains requiring more explicit acknowledgment of uncertainty.

### 3.4. Methodological Confidence in Included Systematic Reviews

Among the 36 systematic and scoping reviews included, overall methodological confidence according to AMSTAR-2 was predominantly critically low (72.2%) or low (27.8%) (Table S3, Supplementary File). Most reviews clearly defined a research question, reported a protocol, and used an adequate multi-database search strategy. However, duplicate data extraction was inconsistently performed, reporting of funding sources for included studies was rare, and only a minority evaluated publication bias or explicitly incorporated risk of bias into the interpretation of results. Confidence also varied across procedural subgroups. Reviews focused on forehead contouring or full facial feminization demonstrated relatively stronger adherence to protocol registration, search comprehensiveness, and bias assessment, whereas reviews on chondrolaryngoplasty or rhinoplasty showed weaker reporting of protocols and synthesis methods. Reviews addressing upper-third or hard-tissue procedures generally performed better on key methodological domains than those focused on lower-third or mixed-topic procedures.

## 4. Discussion

This review provides a structured synthesis of the published FGCS literature and characterizes how the field has developed across procedures, specialties, and outcome domains. A recent single-database review [21] reached convergent conclusions regarding fragmentation of outcome reporting in FGAS. However, the present mapping extends this work through a five-database, language-inclusive search, prospective protocol registration, and formal appraisal of secondary evidence. Among the 338 included studies, most focused on feminizing, upper-third, and hard-tissue procedures, whereas masculinizing, full-face, and soft-tissue procedures were less frequently reported, with emphasis on technique, assessment, and complications, and much less consistent attention to patient-centered domains such as satisfaction, preference, and recovery, as well as broader structural issues such as coverage and ethics. The predominance of technical notes, descriptive cohorts, and case series likely reflects a formative stage of the field, in which procedural codification precedes more comparative or hypothesis-driven research. These contributions remain valuable because they systematize operative knowledge and improve reproducibility of complex FGCS procedures. Importantly, the patterns identified in this map reflect publication trends rather than the actual frequency with which procedures are performed in clinical practice [9].

This imbalance is especially relevant in FGCS because the face plays a central role in gender attribution, social interaction, and self-perception [4]. Although the literature increasingly supports the psychosocial value of these procedures, limited reporting of patient-centered outcomes may reduce the extent to which current evidence can inform preoperative counseling, expectation management, and postoperative assessment in a consistent way [22,23].

### 4.1. Clinical Implications

Despite the relatively limited attention to patient-centered domains in the mapped literature, FGCS has been associated with improvements in gender congruence [24,25,26,27,28,29,30], psychosocial well-being [31,32,33], and quality of life [34,35,36,37,38,39,40,41,42]. However, the present synthesis shows that these outcomes have been evaluated inconsistently and only infrequently through validated PROMs or structured psychological assessments. For surgeons, this creates a practical gap between what can be technically performed and what can be meaningfully discussed, predicted, and evaluated from the patient perspective. As a result, discussions about expected changes in facial congruence or satisfaction after specific procedures often rely more on individual surgeon experience than on comparable, instrument-based evidence. Recent efforts to address this limitation include the development and validation of the GENDER-Q, a PROM created with direct input from transgender and gender-diverse individuals across multiple countries [43]. With 55 independently functioning scales, it offers a structured and culturally adaptable way to assess body image, treatment satisfaction, and health-related quality of life in gender-affirming care [44,45]. FACE-Q and related facial outcome instruments may also complement procedure-specific and aesthetic evaluation [46,47]. Greater incorporation of validated PROMs would improve comparability across studies and make the literature more useful for preoperative counseling, shared decision-making, and postoperative evaluation. In this sense, the evolution of FGCS appears to be parallel to other surgical fields, such as development of core outcome set initiatives to standardize reporting and align research with patient priorities [48]. These findings suggest that FGCS remains in a pre-standardization phase.

Another clinically relevant finding was the limited representation of explicitly multidisciplinary pathways in the published literature [49]. Although multidisciplinary care is widely recommended in transgender health, it remained relatively infrequent in surgical reporting. For surgeons, this matters because patient selection, expectation alignment, perioperative support, and postoperative adaptation may all influence satisfaction and perceived success, particularly in patients with more complex psychosocial or mental health histories [50]. More explicit reporting of multidisciplinary assessment and support pathways would therefore improve interpretation of postoperative satisfaction, recovery reporting, and longer-term patient-reported outcomes. Similarly, the limited attention to patient education, preference elicitation, revision pathways, and postoperative psychosocial support [51] suggests that the field would benefit from moving beyond technical description alone toward more comprehensive and reproducible models of surgical reporting [52,53,54].

A third implication emerges from the appraisal of the 36 SRs included in this map: all were rated as critically low (72%) or low (28%) confidence by AMSTAR-2, with consistent shortfalls in duplicate data extraction, reporting of funding sources of primary studies, and explicit incorporation of risk of bias into synthesis. This finding is clinically relevant because secondary evidence in FGCS is increasingly cited in clinical guidance, policy documents, and insurance determinations, yet rests on syntheses of limited methodological maturity. For surgeons and decision-makers, this means that the apparent consolidation of FGCS evidence through SR should be interpreted with caution until methodological standards in this literature improve. Future syntheses in FGCS should prioritize prospective protocol registration, dual independent extraction, and structured risk-of-bias integration to strengthen the reliability of conclusions drawn for clinical and policy use. When systematic reviews in a field carry such ratings, the practical implication is that pooled statements about FGCS outcomes, including those that may be cited in clinical guidelines, consensus documents, or insurance coverage determinations, should be read as provisional rather than definitive.

The concentration of evidence in North America and Europe, which together account for more than 90% of included studies, likely reflects more than research productivity alone. It is shaped by insurance or public coverage, multidisciplinary centers, surgical volume, and established referral pathways, all of which facilitate systematic outcome reporting. Where FGCS remains self-funded, classified as cosmetic, or delivered by few providers, outcomes are less likely to be documented and published. As a result, current procedural and outcome patterns may partly reflect the priorities and reimbursement structures of high-income health systems rather than universal clinical trends. Low- and middle-income settings remain underrepresented, limiting the transferability of findings. Future reporting should capture health-system context, funding mechanisms, and access pathway alongside clinical outcomes.

Building on these implications, future FGCS research should prioritize three directions: standardized incorporation of validated PROMs (including condition-specific tools such as GENDER-Q and FACE-Q); explicit reporting of multidisciplinary processes, recovery, and revision pathways; and improved methodological standards for secondary evidence. Further work should also examine how factors such as age, gender identity, neurodiversity, trauma history, and sociocultural context shape expectations, experience, and postoperative outcomes [55], and how outcomes vary across health-system contexts, including low- and middle-income settings where access barriers and referral pathways differ substantially.

### 4.2. Strengths and Limitations

This study has several strengths. It synthesized a large body of literature, used a prospectively registered protocol (Table S4), and applied a predefined framework to classify studies across facial region, tissue type, specialty participation, and outcome domains. In addition, included SRs were appraised with AMSTAR-2, allowing secondary evidence to be interpreted in light of its methodological maturity. Its limitations should also be acknowledged. The mapped literature was dominated by observational and descriptive designs, outcome definitions were heterogeneous, and very few studies used validated instruments to assess satisfaction, gender congruence, or psychosocial adjustment [56]. The predominance of studies on feminizing procedures may also reflect historical and institutional differences in access to care or funding priorities, which may limit generalizability across the spectrum of gender-diverse individuals [57]. Geographically, the literature was strongly concentrated in North America and Europe, with limited representation from low- and middle-income settings, where access barriers, referral pathways, and sociocultural contexts may differ substantially [58]. As with any mapping review, the present study describes how evidence has been published rather than whether specific procedures are overused, underused, or superior in practice. Nevertheless, this approach provides a structured view of the field’s current maturity and blind spots and may help guide future evidence synthesis and more standardized reporting across FGCS studies.

## 5. Conclusions

Procedural reporting in FGCS has developed asymmetrically relative to standardized patient-centered outcome measurement, a pattern consistent with the formative stages observed in other surgical fields. For surgeons, this limits comparability across procedures and weakens the evidence base for counseling, multidisciplinary planning, and postoperative evaluation. Future research should prioritize more consistent and validated patient-reporting frameworks.

## Figures and Tables

**Table 1:** Structured classification framework for the FGCS literature.

**Population**	Individuals undergoing FGCS, including transgender women, transgender men, and gender-diverse individuals.
**Intervention**	Surgical procedures targeting facial feminization or masculinization involving hard and/or soft tissue modification. These include, but are not limited to: frontal sinus reduction, orbital rim reshaping, brow lift, hairline advancement, zygomatic augmentation or reduction, rhinoplasty, mandibular contouring, genioplasty, gonial angle reshaping, chin surgery, lip lift, malar augmentation, and chondrolaryngoplasty. Procedures are categorized by facial third (upper, middle, lower) and tissue type (bone or soft tissue), and may be considered as partial FGCS, combined FGCS, or as a full FGCS (F-FGCS).
**Comparison**	Not applicable. No formal comparator is required, as this is a mapping review. However, descriptive comparisons between feminization and masculinization approaches, or between typical male and female anatomical features, may be included when relevant.
**Outcomes**	The primary outcome of this mapping review is the identification and categorization of surgical procedures involved in FGCS, grouped by facial third (upper, middle, lower) or categorized as partial vs. F-FGCS. Secondary outcomes include: Tissue type addressed in the intervention (hard, soft or both tissue types) Surgical specialty or team composition performing the procedures (e.g., oral and maxillofacial surgery, plastic surgery, otolaryngology–head and neck surgery, or interdisciplinary teams). Patient-reported outcome measures (PROMs), such as FACE-Q, visual analog scales (VAS), or other tools assessing satisfaction, aesthetic outcomes, or quality of life. Surgical complications were extracted as reported by each study and subsequently recategorized into standardized groups, including general (e.g., systemic infection, thromboembolism), local (e.g., hematoma, wound dehiscence), functional (e.g., sensory or motor nerve injury, nasal obstruction), aesthetic (e.g., contour irregularities, hypertrophic scarring), and reintervention (e.g., revision surgery), to enable cross-study comparison. When severity grading (e.g., Extended Clavien–Dindo classification of surgical complications) was provided, it was preserved; otherwise, classification was based on the complication description. Economic variables, including reported surgical costs, health system perspectives (when applicable), and the presence of formal economic evaluations (e.g., cost-effectiveness, cost-utility, or cost-analysis studies). Reported surgical outcomes, including objective metrics (e.g., anthropometric or cephalometric analysis, photographic evaluation) and clinician-reported results. Epidemiological patterns, including which procedures are more frequently reported in feminization versus masculinization contexts, geographic region of the studies, patient demographics, and publication year.
**Type of studies**	Original clinical studies (case reports, case series, retrospective and prospective cohort studies, and non-randomized or randomized clinical trials); systematic reviews and meta-analyses; economic evaluations (full cost-effectiveness or cost-utility analyses, or partial evaluations such as cost descriptions or comparisons); technical notes and surgical technique articles with sufficient anatomical and procedural detail; and mixed-methods or qualitative studies reporting PROMs or patient perspectives on specific interventions. Conference abstracts or the gray literature were considered if they provided unique procedural or epidemiological data not available elsewhere and were linked to a full-text source or contactable authorship.
**Data extraction**	Country and study design. Gender target: feminization, masculinization, or procedures applicable to both. Tissue type: hard tissue, soft tissue, or combined approaches. Surgical specialty: plastic surgery, oral and maxillofacial surgery (OMFS), ear–nose–throat/head and neck surgery (ENT-HNS), multi-specialty teams, or other. Terminology was standardized according to professional societies to avoid misclassification across specialties. Diagnosis and planning tools: clinical assessment, 2-dimensional imaging, 3-dimensional imaging, documented patient preference, and patient-education resources used for shared decision-making. Surgical aspects: operative technique descriptions, objective (instrument-based) assessments, revision surgery, and reports on surgical training or formation. Post-surgical aspects: complications, patient-reported outcome measures (PROMs) related to satisfaction, and recovery indicators. Health-system considerations: economic evaluations, insurance coverage issues, and ethical discussions explicitly addressed by the authors.

**Table 2:** Summary of principal evidence concentrations and gaps across facial regions and outcome domains in FGCS.

Facial Region	Well-Represented Domains	Underrepresented/Sparse Domains	Principal Gap for Future Research
**Upper third (forehead, brow, hairline)**	Surgical technique, imaging-based planning, clinical assessment; feminizing, hard-tissue, plastic surgery	Patient preference, recovery, masculinizing equivalents	Link well-characterized skeletal changes to validated patient-reported congruence and satisfaction Report on multidisciplinary, structural and economic dimensions of combined care Develop a facial-specific core outcome set and higher-quality syntheses
**Middle third (malar, nose)**	Surgical technique, aesthetic assessment (rhinoplasty)	Overall study volume low; satisfaction, complications, masculinizing data sparse	
**Lower third (mandible, chin)**	Surgical technique, complications, objective contour measurement; relatively more masculinizing data	Revision pathways, functional outcomes (chondrolaryngoplasty/voice)	
**Multi-region/full-face**	Broadest range of reported outcomes, including satisfaction and Quality of Life	Multidisciplinary pathways, economics, ethics, education still infrequent	
**Cross-cutting (all regions)**	Technique and complication reporting	Insurance (7%), ethics (3%), recovery (2%), economic evaluation; methodologically robust SRs absent	

## Data Availability

The data that support the findings of this study are available from the corresponding author, ELF, upon reasonable request.

## Supplementary Materials

The following supporting information can be downloaded at: https://www.mdpi.com/article/10.3390/medicina62071303/s1, Table S1: Search queries used in the systematic review; Table S2: Excluded studies and reasons; Table S3: Confidence assessment of the results of the included systematic reviews; Table S4: PRISMA 2020 Checklist.

## Abbreviations

The following abbreviations are used in this manuscript:

FGCS	Facial Gender Confirmation Surgery
GD	Gender Dysphoria
GI	Gender Incongruence
SR	Systematic Review
TGD	Trans- and Gender-Diverse
WPATH	World Professional Association for Transgender Health
