# Supplementary material for: Facial Gender-Confirmation Surgery: A Systematic Mapping Review of Surgical Growth and Outcome Gaps
Source: Medicina (Kaunas). 2026 Jul 6;62(7):1303. doi: 10.3390/medicina62071303 (PMC13413436; doi:10.3390/medicina62071303)
Supplement: Supplementary file 1 [file medicina-62-01303-s001.zip › medicina-4382459-supplementary.pdf]

Table S1. Search queries used in the systematic review

Database	Search Query	Filters	Results
**EBSCOhost**	(surg* OR operat* OR procedur* OR intervention* OR implant*) AND (maxillofacial OR craniofacial OR cranio-maxillofacial OR orthognathic OR mandibul* OR maxill* OR jaw OR jawline OR facial OR face OR zygomat* OR midfac* OR forehead OR front* OR hairline OR scalp OR "forehead contour*" OR “orbit*” OR "orbital contour*" OR canth* OR “brow” OR “upper fac*” OR "mandibular contour*" OR "jawline contour*" OR "mandibular angle*" OR “goni*” OR genioplast* OR mentoplast* OR "chin" OR rhinoplast* OR septoplast* OR "nasal reconstruct*" OR chondrolaryngoplast* OR lip OR cheek OR malar) AND ("gender affirm*" OR "gender confirm*" OR "gender reassign*" OR "sex reassign*" OR "gender chang*" OR "gender transit*" OR feminization OR masculinization OR transfemin* OR transmascul*)	None	726
**LiLACS (Plus collection)**	(surg* OR operat* OR procedur* OR intervention* OR implant*) AND (maxillofacial OR craniofacial OR cranio-maxillofacial OR orthognathic OR mandibul* OR maxill* OR jaw OR jawline OR facial OR face OR zygomat* OR midfac* OR forehead OR front* OR hairline OR scalp OR "forehead contour*" OR “orbit*” OR "orbital contour*" OR canth* OR “brow” OR “upper fac*” OR "mandibular contour*" OR "jawline contour*" OR "mandibular angle*" OR “goni*” OR genioplast* OR mentoplast* OR "chin" OR rhinoplast* OR septoplast* OR "nasal reconstruct*" OR chondrolaryngoplast* OR lip OR cheek OR malar) AND ("gender affirm*" OR "gender confirm*" OR "gender reassign*" OR "sex reassign*" OR "gender chang*" OR "gender transit*" OR feminization OR masculinization OR transfemin* OR transmascul*)	None	55
**PubMED**	(surg* OR operat* OR procedur* OR intervention* OR implant*) AND (maxillofacial OR craniofacial OR cranio-maxillofacial OR orthognathic OR mandibul* OR maxill* OR jaw OR jawline OR facial OR face OR zygomat* OR midfac* OR forehead OR front* OR hairline OR scalp OR "forehead contour*" OR “orbit*” OR "orbital contour*" OR canth* OR “brow” OR “upper fac*” OR "mandibular contour*" OR "jawline contour*" OR "mandibular angle*" OR “goni*” OR genioplast* OR mentoplast* OR "chin" OR rhinoplast* OR septoplast* OR "nasal reconstruct*" OR chondrolaryngoplast* OR lip OR cheek OR malar) AND ("gender affirm*" OR "gender confirm*" OR "gender reassign*" OR "sex reassign*" OR "gender chang*" OR "gender transit*" OR feminization OR masculinization OR transfemin* OR transmascul*)	Nonr	1303
**Scopus**	TITLE-ABS-KEY((surg* OR operat* OR procedur* OR intervention* OR implant*) AND (maxillofacial OR craniofacial OR cranio-maxillofacial OR orthognathic OR mandibul* OR maxill* OR jaw OR jawline OR facial OR face OR zygomat* OR midfac* OR forehead OR front* OR hairline OR scalp OR "forehead contour*" OR “orbit*” OR "orbital contour*" OR canth* OR “brow” OR “upper fac*” OR "mandibular contour*" OR "jawline contour*" OR "mandibular angle*" OR “goni*” OR genioplast* OR mentoplast* OR "chin" OR rhinoplast* OR septoplast* OR "nasal reconstruct*" OR chondrolaryngoplast* OR lip OR cheek OR malar) AND ("gender affirm*" OR "gender confirm*" OR "gender reassign*" OR "sex reassign*" OR "gender chang*" OR "gender transit*" OR feminization OR masculinization OR transfemin* OR transmascul*))	None	1046
**Web of Science**	TS=((surg* OR operat* OR procedur* OR intervention* OR implant*) AND (maxillofacial OR craniofacial OR cranio-maxillofacial OR orthognathic OR mandibul* OR maxill* OR jaw OR jawline OR facial OR face OR zygomat* OR midfac* OR forehead OR front* OR hairline OR scalp OR "forehead contour*" OR “orbit*” OR "orbital contour*" OR canth* OR “brow” OR “upper fac*” OR "mandibular contour*" OR "jawline contour*" OR "mandibular angle*" OR “goni*” OR genioplast* OR mentoplast* OR "chin" OR rhinoplast* OR septoplast* OR "nasal reconstruct*" OR chondrolaryngoplast* OR lip OR cheek OR malar) AND ("gender affirm*" OR "gender confirm*" OR "gender reassign*" OR "sex reassign*" OR "gender chang*" OR "gender transit*" OR feminization OR masculinization OR transfemin* OR transmascul*))	None	962

Table S2. Excluded studies and reasons

Reference	Reason
Berli JU, Capitán L, Simon D, Bluebond-Langner R, Plemons E, Morrison SD. Facial gender confirmation surgery—review of the literature and recommendations for Version 8 of the WPATH Standards of Care. *Int J Transgend Health*. 2017;18(3):264-270.	Abstract only
Goldenberg M. Addressing the mental health needs of transgender and gender diverse adult patients seeking facial surgery. *Oral Maxillofac Surg Clin North Am*. 2024;36(2):143-149.	
Lundgren TK, Isung J, Rinder J, Dhejne C, Arver S, Holm LE, Farnebo F. Moving transgender care forward within public health organizations: inclusion of facial feminizing surgery in the Swedish national treatment recommendations. *Arch Sex Behav*. 2016;45(8):1881-1881.	
Pavlidis L, Spyropoulou GA, Dionyssiou D, Demiri E. Full facial feminization surgery: patient satisfaction assessment based on 180 procedures involving 33 consecutive patients. *Plast Reconstr Surg*. 2016;138(4):765e-766e.	
Mukerji S, Debryn D, Price RK, Kim E. Increased incidence of PONV in transgender patients following facial feminization surgery. *Anesth Analg*. 2021;132(3):681-683.	Duplicated study
Greenstein A, Morrison R. Audit to assess the impact on quality of life and delivery of care for patients undergoing facial feminisation. *Br J Oral Maxillofac Surg*. 2014;52(9):e79-e79.	Not retrieved

Gulati A, Soroudi D, Patel NN, Debusk WT, Stephens E, Seth R, Knott PD, Park AM. Postoperative opioid use in staged gender-affirming facial surgery. *Fac Plast Surg Aesthet Med*. 2024;26(7):652-653.	Wrong **P**opulation
Kuperstock JE. Getting to yes: navigating the insurance gauntlet. *Fac Plast Surg Clin North Am*. 2023;31(3):371-374.	
Weinstein B, Schechter L. Wound healing complications in gender-affirming surgery. *Neurourol Urodyn*. 2023;42(4):990-995.	
Price RK, Debryn D, Mukerji S, Nurhussien L, Kim E. Absence of venous thromboembolism in transgender male-to-female patients following the gender-affirming procedure of facial feminization: a retrospective study of 236 patients using hormone therapy. *Anesth Analg*. 2021;132(3):676-677.	Wrong **I**ntervention
Van den Bosch MF, Wiepjes CM, Den Heijer M, Schoonmade LJ, Jonkman REG, Meursinge Reynders RA. Mapping evidence on the effects of gender-affirming hormone therapy on the hard and soft tissues of the craniofacial complex in transgender people: a protocol for a scoping review. *Syst Rev*. 2021;10:1-8.	
Nguyen AT, Li RA, Galiano RD. A critical assessment of online patient education materials for gender-affirming surgery: a systematic review and meta-analysis. *J Sex Med*. 2025;22(7):951-960.	
Facial cosmetic surgery. *J Oral Maxillofac Surg*. 2023;81(7):E300-E324.	Wrong type of study
Balaji, SM: Facial feminization. Ann. Maxillofac. Surg. 6:158. 158,. 2016.	
Bared A, Epstein JS. Gender-affirmation hair transplantation techniques. *Fac Plast Surg Clin North Am*. 2023;31(3):375-380.	
Bared A, Epstein JS. Hair transplantation techniques for the transgender patient. *Fac Plast Surg Clin North Am*. 2019;27(3):227-232.	
Debryn D, Kim E, Mukerji S, Price RK. Incidence of unanticipated airway events in male-to-female transgender patients undergoing facial feminization procedures. *Anesth Analg*. 2021;132(1):27-28.	
Eble D, Hem E. Surgical standards of care and insurance authorization of gender-affirming facial surgery. *Oral Maxillofac Surg Clin North Am*. 2024;36(2):161-169.	
Esmonde N, Najafian A, Penkin A, Berli JU. The role of facial gender confirmation surgery in the treatment of gender dysphoria. *J Craniofac Surg*. 2019;30(5):1387-1392.	
Gutiérrez Santamaría J, Simon D, Capitán L, Pérez de Perceval Tara M, Masiá Gridilla J. Combined orthognathic surgery and jaw contouring to improve long face stigma: lessons learned from facial feminisation. *Br J Oral Maxillofac Surg*. 2025;63(3):330-332.	
Haveles CS, Wang MM, Arjun A, Zaila KE, Lee JC. Effect of cross-sex hormone therapy on venous thromboembolism risk in male-to-female gender-affirming surgery. *Ann Plast Surg*. 2021;86(1):109-114.	
Jaumotte M, Piette E, Walhin N, Le Clercq M, Nizet JL, Gilon Y. Évolution de la demande transgenre en chirurgie maxillo-faciale: le CHU de Liège comme centre de référence. *Rev Med Liege*. 2022;77(10):505-509.	
Li X, Luo Y, Zhang Y. Visualization mapping and current trends of facial contouring procedures: a bibliometric analysis based on Web of Science. *J Craniofac Surg*. 2024;35(4):1346-1351.	
Saikali LM, Herrera CD, Chen AT, Rossi L, Conlon T, Plana NM, Lin IC, Swanson JW, Goldshore M, Morris JB, Guerra CE, Morales CZ. The Center for Surgical Health (CSH): a surgical equity intervention in plastic surgery. *Ann Plast Surg*. 2025;94(5S Suppl 5):S390-S394.	
Spiegel JH. Challenges in care of the transgender patient seeking facial feminization surgery. *Fac Plast Surg Clin North Am*. 2008;16(2):233-238.	

Uranbey O, Kaygisiz OF, Ayrancı F, Yanik S. Exploring the evolution of facial feminization and masculinization surgery: a bibliometric analysis and visualization study. *Maxillofac Plast Reconstr Surg*. 2024;46:1-11.	

Table S3. Confidence assessment of the results of the included systematic reviews

Year	DOI of the Systematic Review	A MeaSurement Tool to Assess systematic Reviews 2 (AMSTAR-2)																
		1	*2	3	*4	5	6	*7	*8	*9	10	*11	12	*13	14	*15	16	Overall
2016	10.1097/PRS.0000000000002171	Y	N	Y	PY	Y	N	N	PY	N	N	NA	NA	N	Y	NA	Y	Critically low
2019	10.1016/j.jpra.2019.07.002	Y	N	Y	N	N	N	N	Y	N	N	NA	NA	N	PY	NA	Y	Critically low
2019	10.1097/SCS.0000000000005101	Y	N	Y	PY	Y	N	N	PY	N	N	NA	NA	N	Y	NA	Y	Critically low
2020	10.1007/s00266-020-01664-8	Y	PY	Y	Y	Y	Y	N	Y	Y	N	NA	PY	Y	Y	NA	Y	Critically low
2020	10.1089/lgbt.2019.0212	Y	PY	Y	PY	N	N	Y	Y	N	N	NA	NA	N	Y	NA	Y	Critically low
2021	10.1089/trgh.2020.0067	Y	NA	Y	NA	N	N	N	Y	NA	N	NA	NA	N	Y	NA	Y	Critically low
2021	10.1097/PRS.0000000000007682	Y	Y	Y	PY	Y	N	N	Y	N	N	NA	NA	N	Y	NA	Y	Critically low
2021	10.1177/0194599821997734	Y	PY	Y	Y	Y	Y	N	Y	PY	N	NA	NA	N	N	NA	Y	Critically low
2022	10.1007/s11930-021-00323-6	Y	PY	Y	PY	Y	PY	N	Y	Y	N	NA	PY	Y	Y	NA	Y	Low
2022	10.1007/s44186-022-00065-6	Y	PY	Y	PY	PY	PY	N	Y	N	N	NA	PY	N	PY	NA	Y	Critically low
2022	10.1016/j.bjps.2022.02.073	Y	PY	Y	PY	Y	Y	N	Y	PY	PY	NA	Y	Y	PY	NA	Y	Low
2022	10.1055/s-0042-1751021	Y	PY	Y	PY	Y	Y	N	Y	N	N	NA	NA	N	Y	NA	Y	Critically low
2022	10.1080/26895269.2022.2038334	Y	PY	Y	PY	Y	Y	N	Y	Y	PY	NA	NA	Y	Y	NA	Y	Low
2022	10.1097/GOX.0000000000004210	Y	PY	Y	PY	Y	N	N	Y	Y	N	NA	NA	Y	Y	NA	Y	Low
2022	10.1097/PRS.0000000000008668	Y	PY	Y	Y	Y	N	N	Y	N	N	NA	NA	N	Y	NA	Y	Critically low
2022	10.1097/SAP.0000000000002952	Y	PY	Y	PY	Y	N	N	Y	N	N	NA	NA	N	Y	NA	Y	Critically low
2022	10.1097/SLA.0000000000004728	Y	PY	Y	Y	Y	Y	N	Y	PY	N	NA	NA	N	Y	NA	Y	Critically low
2023	10.1001/jamanetworkopen.2023.6425	Y	N	Y	PY	Y	Y	N	PY	N	N	NA	PY	Y	Y	NA	Y	Critically low
2023	10.1016/j.jsurg.2023.08.007	Y	PY	Y	PY	Y	Y	N	Y	Y	N	NA	NA	Y	Y	NA	Y	Low
2023	10.1097/PRS.0000000000010010	Y	PY	N	PY	N	N	N	Y	N	N	N	N	N	N	N	Y	Critically low
2023	10.1097/SCS.0000000000009157	Y	PY	Y	Y	Y	Y	N	Y	N	N	Y	N	N	Y	N	Y	Critically low
2023	10.1177/27325016231170403	Y	PY	PY	PY	Y	Y	N	Y	N	N	NA	NA	N	Y	NA	Y	Critically low
2024	10.1002/ohn.825	Y	Y	Y	PY	Y	Y	N	Y	N	N	NA	PY	Y	Y	NA	Y	Critically low
2024	10.1007/s00266-024-04143-6	Y	PY	PY	N	N	N	N	Y	PY	N	NA	PY	N	N	NA	Y	Critically low
2024	10.1016/j.bjps.2023.12.001	Y	Y	Y	Y	Y	PY	N	Y	PY	N	NA	N	Y	Y	NA	Y	Low
2024	10.1016/j.bjps.2024.01.049	Y	PY	Y	PY	Y	Y	N	Y	Y	N	NA	PY	Y	Y	NA	Y	Low

2024	10.1037/sgd0000702	Y	PY	Y	PY	Y	Y	N	Y	N	N	NA	NA	Y	Y	NA	Y	Critically low
2024	10.1080/26895269.2023.2278736	Y	PY	Y	PY	Y	Y	N	Y	Y	N	Y	Y	Y	Y	N	Y	Critically low
2024	10.1089/fpsam.2021.0293	Y	Y	Y	PY	Y	Y	N	Y	Y	N	NA	NA	Y	Y	NA	Y	Low
2024	10.1089/trgh.2023.0020	Y	PY	Y	PY	Y	Y	N	Y	PY	N	NA	NA	N	Y	NA	Y	Critically low
2024	10.1093/asj/sjae082	Y	PY	Y	PY	Y	Y	N	Y	N	Y	NA	NA	Y	Y	NA	Y	Critically low
2024	10.1097/PRS.0000000000010594	Y	NA	Y	Y	Y	N	N	Y	N	N	NA	NA	N	Y	NA	Y	Critically low
2025	10.1097/SCS.0000000000010840	Y	Y	PY	Y	Y	N	Y	Y	PY	N	Y	N	N	Y	Y	Y	Low
2025	10.1111/ans.70028	Y	PY	Y	PY	Y	Y	N	Y	Y	N	Y	Y	Y	Y	N	Y	Critically low
2025	10.1177/27325016251323096	Y	PY	Y	PY	Y	N	N	Y	N	N	NA	NA	N	Y	NA	Y	Critically low
2025	10.3390/jcm14010182	Y	PY	Y	PY	Y	Y	N	Y	Y	N	NA	NA	Y	Y	NA	Y	Low

N: No; NA: Not aplicable; PY: Partial Yes; Yes.
Note: Columns painted in light blue indicates a critical item in the AMSTAR-2 evaluation

Table S4. PRISMA 2020 Checklist

Section and Topic	Item #	Checklist item	Location where item is reported
**TITLE**			
Title	1	Identify the report as a systematic review.	**Title, lines 2–3**
**ABSTRACT**			
Abstract	2	See the PRISMA 2020 for Abstracts checklist.	**Abstract, lines 24–48**
**INTRODUCTION**			
Rationale	3	Describe the rationale for the review in the context of existing knowledge.	**Introduction, lines 50–80**
Objectives	4	Provide an explicit statement of the objective(s) or question(s) the review addresses.	**Introduction, lines 80–86**

Section and Topic	Item #	Checklist item	Location where item is reported
**METHODS**			
Eligibility criteria	5	Specify the inclusion and exclusion criteria for the review and how studies were grouped for the syntheses.	**Methods, lines 100–104; Table 1**
Information sources	6	Specify all databases, registers, websites, organisations, reference lists and other sources searched or consulted to identify studies. Specify the date when each source was last searched or consulted.	**Methods, lines 106–117**
Search strategy	7	Present the full search strategies for all databases, registers and websites, including any filters and limits used.	**Methods, lines 107–115; Table S1**
Selection process	8	Specify the methods used to decide whether a study met the inclusion criteria of the review, including how many reviewers screened each record and each report retrieved, whether they worked independently, and if applicable, details of automation tools used in the process.	**Methods, lines 117–125**
Data collection process	9	Specify the methods used to collect data from reports, including how many reviewers collected data from each report, whether they worked independently, any processes for obtaining or confirming data from study investigators, and if applicable, details of automation tools used in the process.	**Methods, lines 126–137**
Data items	10a	List and define all outcomes for which data were sought. Specify whether all results that were compatible with each outcome domain in each study were sought (e.g. for all measures, time points, analyses), and if not, the methods used to decide which results to collect.	**Table 1 (Outcomes); Methods, lines 138–148**
	10b	List and define all other variables for which data were sought (e.g. participant and intervention characteristics, funding sources). Describe any assumptions made about any missing or unclear information.	**Table 1 (Data extraction); Methods, lines 136–137**

Section and Topic	Item #	Checklist item	Location where item is reported
Study risk of bias assessment	11	Specify the methods used to assess risk of bias in the included studies, including details of the tool(s) used, how many reviewers assessed each study and whether they worked independently, and if applicable, details of automation tools used in the process.	**Methods, lines 149–154 (AMSTAR-2; adapted)**
Effect measures	12	Specify for each outcome the effect measure(s) (e.g. risk ratio, mean difference) used in the synthesis or presentation of results.	**N/A (mapping review)**
Synthesis methods	13a	Describe the processes used to decide which studies were eligible for each synthesis (e.g. tabulating the study intervention characteristics and comparing against the planned groups for each synthesis (item #5)).	**Methods, lines 138–148**
	13b	Describe any methods required to prepare the data for presentation or synthesis, such as handling of missing summary statistics, or data conversions.	**Methods, lines 139–148**
	13c	Describe any methods used to tabulate or visually display results of individual studies and syntheses.	**Methods, lines 141–145; Figures 3–4**
	13d	Describe any methods used to synthesize results and provide a rationale for the choice(s). If meta-analysis was performed, describe the model(s), method(s) to identify the presence and extent of statistical heterogeneity, and software package(s) used.	**Methods, lines 138–148 (no meta-analysis)**
	13e	Describe any methods used to explore possible causes of heterogeneity among study results (e.g. subgroup analysis, meta-regression).	**N/A (no meta-analysis)**
	13f	Describe any sensitivity analyses conducted to assess robustness of the synthesized results.	**N/A (mapping review)**

Section and Topic	Item #	Checklist item	Location where item is reported
Reporting bias assessment	14	Describe any methods used to assess risk of bias due to missing results in a synthesis (arising from reporting biases).	**N/A; AMSTAR-2, lines 149–151**
Certainty assessment	15	Describe any methods used to assess certainty (or confidence) in the body of evidence for an outcome.	**Methods, lines 149–151 (AMSTAR-2; adapted)**
**RESULTS**			
Study selection	16a	Describe the results of the search and selection process, from the number of records identified in the search to the number of studies included in the review, ideally using a flow diagram.	**Results, lines 155–161; Figure 2**
	16b	Cite studies that might appear to meet the inclusion criteria, but which were excluded, and explain why they were excluded.	**Results, line 161; Figure 2; Table S2**
Study characteristics	17	Cite each included study and present its characteristics.	**Results, lines 164–189; Figure 4**
Risk of bias in studies	18	Present assessments of risk of bias for each included study.	**Results, lines 241–254; Table S3**
Results of individual studies	19	For all outcomes, present, for each study: (a) summary statistics for each group (where appropriate) and (b) an effect estimate and its precision (e.g. confidence/credible interval), ideally using structured tables or plots.	**Figure 4 (online map); adapted (no effect estimates)**
Results of syntheses	20a	For each synthesis, briefly summarise the characteristics and risk of bias among contributing studies.	**Results, lines 164–254; Figures 3–4**
	20b	Present results of all statistical syntheses conducted. If meta-analysis was done, present for each the summary estimate and its precision (e.g. confidence/credible interval) and measures of statistical heterogeneity. If comparing groups, describe the direction of the effect.	**Results, lines 164–240 (descriptive)**

Section and Topic	Item #	Checklist item	Location where item is reported
	20c	Present results of all investigations of possible causes of heterogeneity among study results.	**N/A (no meta-analysis)**
	20d	Present results of all sensitivity analyses conducted to assess the robustness of the synthesized results.	**N/A (mapping review)**
Reporting biases	21	Present assessments of risk of bias due to missing results (arising from reporting biases) for each synthesis assessed.	**N/A; AMSTAR-2, lines 246–247**
Certainty of evidence	22	Present assessments of certainty (or confidence) in the body of evidence for each outcome assessed.	**Results, lines 241–243; Table S3**
**DISCUSSION**			
Discussion	23a	Provide a general interpretation of the results in the context of other evidence.	**Discussion, lines 255–278**
	23b	Discuss any limitations of the evidence included in the review.	**Discussion, lines 334–343**
	23c	Discuss any limitations of the review processes used.	**Discussion, lines 343–345**
	23d	Discuss implications of the results for practice, policy, and future research.	**Discussion, lines 279–328; Conclusions, lines 348–354**
**OTHER INFORMATION**			
Registration and protocol	24a	Provide registration information for the review, including register name and registration number, or state that the review was not registered.	**Methods, lines 89–92; Abstract, line 33**
	24b	Indicate where the review protocol can be accessed, or state that a protocol was not prepared.	**Methods, lines 89–92**

Section and Topic	Item #	Checklist item	Location where item is reported
	24c	Describe and explain any amendments to information provided at registration or in the protocol.	**Methods, line 92**
Support	25	Describe sources of financial or non-financial support for the review, and the role of the funders or sponsors in the review.	**Funding, line 365**
Competing interests	26	Declare any competing interests of review authors.	**Conflicts of Interest, line 370**
Availability of data, code and other materials	27	Report which of the following are publicly available and where they can be found: template data collection forms; data extracted from included studies; data used for all analyses; analytic code; any other materials used in the review.	**Data Availability, lines 368–369; lines 233–236; Suppl. File 1**

From: Page MJ, McKenzie JE, Bossuyt PM, Boutron I, Hoffmann TC, Mulrow CD, et al. The PRISMA 2020 statement: an updated guideline for reporting systematic reviews. BMJ 2021;372:n71. doi: 10.1136/bmj.n71. This work is licensed under CC BY 4.0. To view a copy of this license, visit https://creativecommons.org/licenses/by/4.0/
